# The Role of Circulating Free DNA and MicroRNA in Non-Invasive Diagnosis of HBV- and HCV-Related Hepatocellular Carcinoma

**DOI:** 10.3390/ijms19041007

**Published:** 2018-03-28

**Authors:** Francesca Pezzuto, Luigi Buonaguro, Franco Maria Buonaguro, Maria Lina Tornesello

**Affiliations:** Molecular Biology and Viral Oncology Unit, Istituto Nazionale Tumori IRCCS “Fondazione G. Pascale”, 80131 Napoli, Italy; francesca.pezzuto1987@gmail.com (F.P.); l.buonaguro@istitutotumori.na.it (L.B.); fm.buonaguro@istitutotumori.na.it (F.M.B.)

**Keywords:** liquid biopsy, early diagnosis, circulating free DNA, microRNA, hepatocellular carcinoma, hepatitis B virus, hepatitis C virus, long non coding RNA

## Abstract

Hepatocellular carcinoma (HCC) is the third and the fifth leading cause of cancer related death worldwide in men and in women, respectively. HCC generally has a poor prognosis, with a very low 5-year overall survival, due to delayed diagnosis and treatment. Early tumour detection and timely intervention are the best strategies to reduce morbidity and mortality in HCC patients. Histological evaluation of liver biopsies is the gold standard for cancer diagnosis, although it is an invasive, time-consuming and expensive procedure. Recently, the analysis of circulating free DNA (cfDNA) and RNA molecules released by tumour cells in body fluids, such as blood serum, saliva and urine, has attracted great interest for development of diagnostic assays based on circulating liver cancer molecular biomarkers. Such “liquid biopsies” have shown to be useful for the identification of specific molecular signatures in nucleic acids released by cancer cells, such as gene mutations and altered methylation of DNA as well as variations in the levels of circulating microRNAs (miRNAs) and long non-coding RNAs (lncRNAs). Body fluids analysis may represent a valuable strategy to monitor liver disease progression in subjects chronically infected with hepatitis viruses or cancer relapse in HCC treated patients. Several studies showed that qualitative and quantitative assays evaluating molecular profiles of circulating cell-free nucleic acids could be successfully employed for early diagnosis and therapeutic management of HCC patients. This review describes the state of art on the use of liquid biopsy for cancer driver gene mutations, deregulated DNA methylation as well as miRNA levels in HCC diagnosis.

## 1. Introduction

Primary liver cancer represents the sixth most common and deadly tumour in the world with 782,000 new cases and 746,000 deaths in 2012 [1]. Hepatocellular carcinoma (HCC) is the major histological subtype accounting for 85% of all liver cancer cases worldwide [2,3,4]. The major risk factors for the development of HCC are hepatitis B (HBV) and hepatitis C (HCV) chronic infections which have been found to be associated with 56% and 20% of cases, respectively [5]. HBV-related HCC is more frequent (67%) than HCV-related HCC (12%) in less developed countries, while HBV-related HCC is less frequent (23%) than HCV-related tumours (44%) in more developed countries [5]. The HBV and HCV viral proteins along with biological and environmental co-factors promote chronic insult to hepatocytes, accumulation of genetic damages and epigenetic deregulation, which cause over the time, the hepatic damage, cirrhosis, fibrosis and cancer [6].

The diagnosis of liver cancer is generally performed by imaging techniques, such as ultrasonography, computed tomography and magnetic resonance tomography, in combination with the dosage of plasmatic alpha-fetoprotein (AFP) and histological analysis of tissue biopsies [7]. The diagnostic imaging methods have the advantage of not being invasive and the disadvantage of insufficient sensitivity for detection of HCC nodules smaller than one cm [8]. The measurement of AFP in the blood, which is one of the most widely used screening tests to diagnose HCC, has a limited sensitivity and specificity given that some liver nodules may not release AFP, and patients with chronic active hepatitis or liver cirrhosis may have high levels of AFP [9]. To date, liver tissue biopsy is considered the gold standard for HCC diagnosis but has drawbacks of invasiveness, is effective when the nodule has reached considerable dimensions and carries the risk of neoplastic cells diffusion [10]. 

The treatment options for HCC include surgical resection, transarterial chemoembolization, radiofrequency ablation, high-intensity focused ultrasound, targeted molecular therapy such as sorafenib and more rarely liver transplantation. The success of these treatments could be seriously improved by early cancer detection and effective post-treatment monitoring [11].

Recent studies have shown that specific biomarkers of cancer cells are detectable in the body fluids such as blood serum, urine and saliva, which for this reason have been termed “liquid biopsies”. The blood serum contains detectable amounts of circulating free DNA (cfDNA) ranging from 1 to 500 ng/mL, showing the mutational spectrum of the tumour cell DNA [11]. In addition, cfDNA fragments have the same methylation profile as the original tumour DNA, suggesting the possibility of analyzing the cfDNA methylation status for monitoring tumour growth. Many tumour cells, including liver cancer cells, release specific microRNAs (miRNAs) and long non-coding RNAs (lncRNAs) in the bloodstream, either as free molecules or entrapped in vesicles such as exosomes [12,13,14]. Such molecules may represent important biomarkers of tumour development.

We performed a systematic review of published studies to investigate the state of art on the employment of screening tests based on circulating liver biomarkers for diagnosis and prognosis of HCC associated with different aetiologies (Table 1).

Published data were searched in Medline using the terms (“hepatocellular” OR “Liver” AND “Cancer”) AND (“liquid biopsy” OR “blood” OR “plasma” OR “serum” OR “urine”) AND (“circulating free DNA”) AND (“microRNA OR miRNA”) AND (“DNA mutations”) AND (“DNA methylation”) AND (“microsatellite instability”) AND (“microRNA” OR “miRNA”) AND (“long non coding RNA” OR “lncRNA”) AND (“extracellular vesicles” OR “exosomes”). The search was updated on 28 January 2018.

## 2. Circulating Free DNA

Circulating free DNA was first described by Mandel and Metais in 1948 [15]. Thirty years later, Leon et al. observed that the amount of cfDNA was higher in cancer patients compared to healthy controls and that its concentration in the serum further increased after radiation therapy [16,17,18]. Nowadays, it is recognized that cfDNA originates mainly from the activity of macrophages or other scavenger cells which engulf apoptotic and necrotic tumour cells and release digested tumour DNA into the blood stream [19,20]. Because the length of digested DNA molecules is around 160 bp, the recovery and analysis of cfDNA requires highly sensitive techniques [21]. 

Qualitative and quantitative analysis of cfDNA as a diagnostic and prognostic parameter in cancer patients has been studied by many groups. Piciocchi et al. observed higher levels of cfDNA among patients affected by HCC, cirrhosis and HCV-related chronic hepatitis compared to healthy subjects, and the increase was directly correlated to the disease status and reduced patients’ survival [22]. Other studies, however, reported wide inter-subject variations in cfDNA levels, showing sometimes overlapping values between malignant and benign diseases or healthy controls. In addition, patients affected by some non-oncologic pathologies such as autoimmune diseases are also characterized by increased levels of cfDNA in the peripheral blood, making this parameter not specific for cancer diagnosis [23,24]. 

Moreover, the different methodologies of cfDNA extraction may bias its quantification given that different extraction kits with variable recovery efficiencies can hamper the measurement of the real cfDNA levels in the blood serum [36,37]. 

Several studies analyzed cfDNA integrity as a parameter of the disease status although with contrasting results [38]. Huang et al. reported low integrity of cfDNA in a cohort of Chinese HCC patients, mainly related to HBV infection, compared to patients with benign liver disease and healthy subjects [39]. Interestingly, the cfDNA integrity test had a sensitivity, specificity and accuracy of 43.4%, 100% and 60%, respectively, in the detection of HCC [39]. The high efficacy of cfDNA integrity as a diagnostic marker was achieved by the improved sensitivity of PCR protocols based on short amplicons targeting the notably short tumour derived DNA fragments [40,41]. Conversely, Wang et al. reported that the increased cfDNA integrity was associated with cancer, and measurement of this parameter may be useful for cancer detection [42]. Accordingly, two other studies observed that cfDNA integrity was significantly higher in HCC patients compared to HBV- and HCV-positive patients and healthy controls [41,43]. Elshimali et al. also observed that cfDNA integrity was associated with tumour size, TNM stage, vascular invasion, lymph node involvement, distant metastasis and poor survival [36].

**Figure 1:**
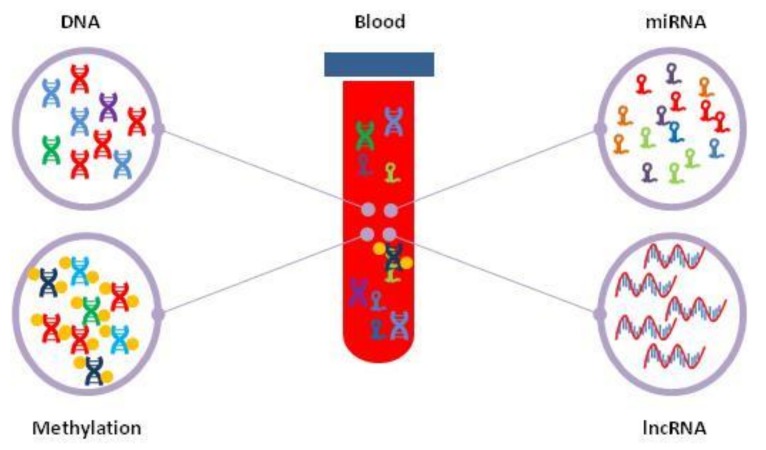
Schematic representation of the liquid biopsy as tool for the analysis of circulating DNAs and RNAs released from apoptotic or necrotic cancer cells into the blood stream.

The majority of cancer types are characterized by distinctive somatic mutations which can be identified in the DNA released by cancer cells and, in combination with the measurement of cfDNA levels, may provide valuable clinical information, Figure 1 [44]. Several methodologies, mainly based on the polymerase chain reaction (PCR) technique, have been used to detect tumour-related known mutations by specific probes in cfDNA including the amplification refractory mutation system (ARMS) PCR, single-strand conformation polymorphism (SSCP), mutant enriched (ME) PCR, mutant allele specific amplification (MASA), pyrophosphorolysis-activated polymerization allele specific (PAP-A) PCR, and restriction fragment length polymorphism (RFLP-PCR) [45]. In addition, novel methods based on digital technology have been introduced in cfDNA analysis such as the droplet digital PCR (ddPCR). This technique is based on a droplet generating system, and BEAMing, involving the use of beads, emulsions, amplification, magnetics, and microfluidics digital PCR [46,47,48,49]. All such PCR-based techniques are very sensitive but have the disadvantage of generating false positive results when the target DNA has a low copy number. Next generation sequencing (NGS) is widely used to analyze large genomic regions on cfDNA and to detect, besides the known tumour related mutations, the less common but clinically relevant variations. However, NGS with its high degree of sensitivity may originate false positive results which require careful validation of all steps involved in the experimental procedures including blood collection, cfDNA extraction, library preparation, sequencing and variant callings [50]. 

Many studies have been published on the detection of tumour-specific somatic mutations in cfDNA of various cancer types [51]. A significant association has been reported between tumour stage and cancer-related genetic alterations, such as nucleotide changes in *TP53*, *KRAS*, *APC* and allelic imbalances, in the blood of patients affected by breast, ovarian, pancreatic, colorectal cancer and oral carcinoma as well as HCC [52,53,54]. Cancer driver mutations in TP53 and CTNNB1 genes as well as in the TERT promoter region have been frequently identified in tumour tissues of HCC patients [55,56]. These mutations have been also detected in the peripheral blood of liver cancer patients. Particularly, Huang et al. analyzed the mutational profile of TP53 (c.747 G > T), CTNNB1 (c.121A > G, c.133 T > C), and TERT promoter (−124 C > T) in 48 HCC cases by digital droplet PCR assay and found that 56.3% of patients had at least one mutation in cfDNA and 22.2% had concordant mutations in tumour DNA and cfDNA [57]. Liao et al. investigated the mutational profile of these three genes in a cohort of Chinese HCC patients and identified TERT, CTNNB1 and TP53 mutations in 4.9%, 9.8% and 4.9%, respectively, of serum samples [26]. Interestingly, one patient had the CTNNB1 mutation (c.122 C > T) in cfDNA but not in the primary tumour DNA, suggesting that circulating DNA fragments originated from different tumour nodules with heterogeneous DNA alterations [26]. However, discordant mutations between the DNA from primary tumour and cfDNA could also indicate the occurrence of false positive results generated by highly sensitive techniques and repeated experiments are needed to rule out such a possibility. 

Dietary exposure to aflatoxin B1 (AFB1) in Asia and Africa, in association with HBV infection, has shown to increase the risk of HCC. The AFB1-related HCC patients frequently have distinctive mutations in TP53 gene, such as the G to T transversion at codon 249 causing the arginine substitution to serine (R249S) [58]. Jiao et al. identified the TP53 R249S mutation in 7.3% of HCC from Hispanic patients living in South Texas but not among 218 HCC non-Hispanic patients and not in 96 subjects with advanced fibrosis or cirrhosis living in the same region, suggesting that AFB-1 exposure may have occurred only in the Hispanic population [59]. They observed that patients with TP53 R249S mutations were significantly younger and had a lower overall survival. In Gambia, a country with high exposure to AFB-1, the TP53 R249S mutation has been identified in 35% of HCC biopsies and in 42% of plasma samples from HCC patients with a concordance of 88.5% between tumour tissues and matched plasma [60]. Moreover, Huang et al. [61] studied the intra tumour genetic heterogeneity in relation to the type of mutations identified in cfDNA fragments by analyzing a large panel of mutations in HCC driver genes, comprising TP53, CTNNB1, PIK3CA and ARID1A. They observed that cfDNA might provide a higher genome profiling potential than a single tumour specimen using highly sensitive deep sequencing technology [61]. More recently, Cohen et al. developed a blood assay able to diagnose the most common mutations in eight cancer types, including HCC, through the analysis of circulating proteins, such as CA19-9, HGF, OPN, TIMP-1, CA-125, CEA, MPO and PRL as well as of genetic alterations in cfDNA, such as the mutations in TP53, CTNNB1, CDKN2A, PTEN and KRAS genes [62]. This a combined test, based on Luminex bead immunoassay technology, showed 100% sensitivity for the detection of cancer lesions in the early stages [62]. Cai et al. performed a whole exome sequencing analysis of DNA extracted from paired biopsies and plasma samples of four HCC patients and showed that 96.9% of the tissue mutations could be also detected in cfDNA [63]. Such results strongly suggest that the analysis of cfDNA could overcome tumour heterogeneity with uneven distribution of mutations in different nodules and could allow rapid evaluation of therapeutic responses in the longitudinal monitoring of treated patients [63]. Furthermore, they found that the valine-to-methione substitution at codon 174 in Hck tyrosine kinase, a recurrent metastasis related mutation, could promote the migration and invasion of HCC cells [63].

High levels of HBV DNA in the blood serum have shown to be a strong risk factor for HCC onset. Chen et al. observed that an elevated HBV DNA level (≥10,000 copies/mL) in the serum is a predictor of HCC independently from HBeAg, alanine aminotransferase level and liver cirrhosis [64]. Moreover, circulating HBV DNA has been suggested to be an early indicator of the success or failure of transarterial chemoembolisation [65].

## 3. DNA Methylation

DNA methylation is one of the most common epigenetic mechanisms used by the cells to control gene expression. It consists of the addition of methyl groups at CpG dinucleotides which are concentrated at specific clusters defined as CpG islands [66]. DNA methylation is usually a repressive mechanism causing specific gene silencing and allele inactivation of the X-chromosome. Aberrant methylation of normally unmethylated 5′-CpG-rich regions in cancer cells leads to the repression of several genes coding for factors involved in DNA damage repair, cell cycle regulation and apoptosis [67]. HBV infection has shown to affect the methylation of several genes including Ras association domain family 1 isoform A (RASSF1A), Glutathione S-Transferase Pi 1 (GSTP1), Cyclin Dependent Kinase Inhibitor 2A (p16[INK4A]), E-cadherin (CDH1) and Cyclin Dependent Kinase Inhibitor 1A (p21[WAF1/CIP1]) genes, while HCV infection has been associated with aberrant methylation of Adenomatous Polyposis Coli (APC), Suppressor of Cytokine Signaling 1 (SOCS-1), Growth Arrest and DNA Damage Inducible Beta (Gadd45β), O-6-Methylguanine-DNA Methyltransferase (MGMT) and Signal Transducer and Activator of Transcription 1 (STAT1) genes [68,69]. Hypermethylation of the RASSF1A gene is frequently observed in HCC [27,33,67]. Chan et al. found the RASSF1A gene hyper methylated in 93% of the sera of HBV-related HCC patients and in 58% of the sera of HBV chronic infected patients suggesting that RASSF1A hypermethylation could represent an early event in HCC pathogenesis [31]. Other studies reported that patients with high RASSF1A methylation at diagnosis or one year after tumour resection show generally poor disease-free survival, suggesting that RASSF1A methylation could be a good cancer prognostic marker [27,32,33]. Conversely, Hui-Chen et al. failed to find RASSF1A gene methylation in plasma of Taiwanese HCC patients although it was hypermethylated in tumour biopsies [70]. Dong et al. reported that several genes, such as RASSF1A, APC, Blood Vessel Epicardial Substance (BVES), Homeobox A9 (HOXA9), GSTP1, and Tissue Inhibitor of Metallopeptidase Inhibitor 3 (TIMP3), were hypermethylated in cancer biopsies of 343 HCC patients but only RASSF1A, BVES, and HOXA9 gene promoters were found significantly hypermethylated also in the sera of these patients [17]. In addition, in this study, the sensitivity of RASSF1A hypermethylation in the serum was higher than AFP (≥20 ng/L) in distinguishing HCC from HBV chronic infected patients [17].

The promoter region of GSTP1, encoding for Glutathione S-transferase P1, has been found to be hyper methylated in about 50% of cancer tissues including HCC [71,72]. The aberrant methylation of GSTP1 has been shown to be associated with HCC progression [1], and to be more frequent in tumours characterized by capsular invasion and metastasis [73]. Several studies suggested GSTP1 methylation as a diagnostic marker for HCC reporting a sensitivity of 50–75% and a specificity of 70–91% with a performance superior to that of APC or RASSF1 genes [28]. The meta-analysis conducted by Liu et al. analyzing the methylation status of GSTP1 in 646 HCC tissues, APC in 592, and SOCS1 in 512 HCC tissues showed a strong correlation between the hypermethylation of such genes and the risk of HCC and suggested such epigenetic alterations as promising biomarkers for HCC development [74]. Huang et al. analyzed the methylation status of GSTP1, RASSF1A, APC and Secreted Frizzled Related Protein 1 (SFRP1) genes in plasma samples of 72 patients with HCC and 37 subjects with benign liver diseases showing that RASSF1A methylation was positively correlated with tumour size, while GSTP1 methylation was associated with elevated AFP levels in the serum, and SFRP1 methylation was more common in females [27]. The authors also found that hypermethylation of all these genes had a sensitivity of 84.7% in the detection of HCC [27]. Wang et al. reported that the methylation status of GSTP1 contributes to hepatic carcinogenesis since this gene has been found hypermethylated in the serum of 50% of HCC patients and in 37.5% of cirrhotic patients [29].

Other hypermethylated genes detected in the plasma of HCC patients are CDKN2A, which encodes for p16, an inhibitor of cyclin D-dependent kinases, and SOCS3, which encodes for the cytokine signaling 3 suppressor [30,34]. Moreover, Han et al. found that G Protein-Coupled Bile Acid Receptor 1 (TGR5), a membrane-bound receptor with a crucial role in regulating bile homeostasis and glucose metabolism, is aberrantly methylated in HCC and could have a diagnostic value of AFP in the discrimination of HCC from HBV chronic infected patients [35]. TGR5 acts as tumour suppressor gene, in fact its activation greatly inhibits the proliferation and migration of human liver cancer cells in vitro while the deficiency of TGR5 enhances chemical-induced liver carcinogenesis [35]. Recently, other genes have been found hypermethylated in HCC, such as AKR1B1, GRASP, MAP9, NXPE3, RSPH9, SPINT2, STEAP4, ZNF154, VIM and FBLN1 genes [75,76]. On the other hand, an elevated level of hypomethylated LINE1 Type Transposase Domain Containing 1 (LINE-1) in the serum has been associated with tumour progression, invasiveness and poor prognosis in HCC patients [77,78,79]. Liu et al. found that LINE-1 was hypomethylated in 66.7% of sera from HCC patients and was associated with HBsAg positivity, tumour size, AFP levels and poor survival [32]. Importantly, measurement of LINE-1 hypomethylation and RASSF1A promoter hypermethylation was found to be significantly correlated with early recurrence and poor prognosis in HCC patients after curative resection [32].

## 4. Microsatellite Instability

Microsatellites are short, highly repeated DNA sequences commonly present in the eukaryotic genomes [80]. Loss and length alteration of microsatellite regions are frequent events in the neoplastic process, suggesting their possible employment in the tumour diagnosis. The comparative genomic hybridization (CGH) technique has enabled the study of some microsatellite alterations in HCC genomes such as those affecting chromosome 8p, 17p and 19p deletions, which might cause HCC metastasis [81]. Moreover, two microsatellite markers located on the chromosome 8p, namely D8S258 and D8S264, have been found to be associated with increased cfDNA levels and involved in HCC progression, metastasis and reduced survival [82]. Pang et al. observed microsatellite instability and loss of heterozygosity of D8S277, D8S298, and D8S1771 located on chromosome 8p in the plasma DNA of HCC patients [83].

The analysis of 109 microsatellite markers, representing 24 chromosomal arms, in 21 cases of HCC, six cholangiocarcinoma and 27 chronic hepatitis or cirrhosis cases performed by Chang et al. showed at least one loss of heterozygosity in the cfDNA of approximately 76% of HCC patients. None of the cholangiocarcinoma patients exhibited loss of heterozygosity, suggesting that microsatellite markers might be appropriate for differential diagnosis of primary liver cancers [84]. Interestingly, 71.4% of HCC patients with AFP levels below 20 ng/mL showed loss of heterozygosity in the microsatellite regions, suggesting that this factor is an early marker of tumour development [84].

## 5. Circulating MicroRNAs

MiRNAs are short non-coding RNAs which regulate gene expression through their binding to the 3′UTR of mRNAs and consequent degradation or translational repression of targeted gene transcripts [85]. Deregulation of miRNAs levels in the cells plays an important role in tumour development.

Numerous miRNAs have shown to be associated with HCC on the basis of their differential expression in tumour versus non-tumour liver tissues such as the miR-122, miR-200a, miR-21, miR-223, let-7f, and miR-155 [85]. The role of circulating miR-122 and let-7 in the early diagnosis of HCC was suggested by the observation that their levels in the sera of HBV positive patients with dysplastic nodules and of early stage HCC patients had a sensitivity comparable to AFP testing [57]. Moreover, the hyper expression of let-7f in the serum has been shown to correlate with tumour size above 5 cm in diameter and with early recurrence [86]. miR223-3p and miR-125b-5p also were evaluated as good biomarkers in HBV-positive HCC [87]. Zheng et al. analyzed the serum levels of miR-125-5p in 120 patients with HCC, 91 with chronic HBV and 164 healthy controls, observing increased expression in liver fibrosis but not in HCC. Low serum levels of miR-125a-5p in HCC patients were correlated with a poor prognosis [88].

miR-122 has been shown to have a major role in HCV-related HCC. Zekri et al, using a panel of miR-122, miR-885-5p, and miR-29b in association with AFP testing, obtained a high diagnostic accuracy for early detection of HCC in a normal population, while using a panel of miR-122, miR-885-5p, miR-221, and miR-22 with AFP, obtained a high diagnostic accuracy for early detection of HCC in cirrhotic patients [89]. In addition, Qu et al., testing for miR-143 and miR-215 in association with AFP, showed a good efficiency in HCC diagnosis [90]. Okajima et al. analyzed the expression of four oncogenic miRNAs, namely miR-151, miR-155, miR-191 and miR-224, in the plasma of 107 HCC patients and 75 healthy volunteers. They observed that miR-224 was highly expressed in HCC tissues and plasma, but the levels decreased significantly following surgery, suggesting that miR-224 reflects tumour dynamic [91]. Similarly, miR-500 has been found to be largely expressed in sera of HCC patients and decreased to normal levels after surgery [92]. The expression profiles of miR-21 showed contrasting results. Ge et al. and Zhuang et al. observed down-regulation of miR-21 in HCC [86,93], while Zhou et al. and Amr et al. found hyper expression of miR-21 in HCC patients [94,95]. Recently, Ding et al. performed a meta-analysis including 24 studies and concluded that the high expression levels of miR-21, as well as miR-122 and miR-199, are highly specific for the diagnosis of HCC [96]. Despite its expression levels, miR-21 has been found to be involved in tumour cell migration, invasion and in metastasis [94,95]. Other miRNAs have been found to be associated with the development of metastasis, including miR-182, which is able to down-regulate metastasis suppressor 1 [97], and miR-331–3P, targeting the PH domain and leucine-rich repeat protein phosphatase [98].

miR-16 is down-regulated in the serum of HCC patients, and the low expression is correlated with some clinical features such as platelets, prothrombin time and bilirubin [86]. miR-30e and miR-223 have also been found at significantly lower levels in the sera of HCC patients compared to chronic liver diseases patients and healthy volunteers [99]. In addition, miR-26a and miR-101 are deregulated in the serum of HCC patients and could be used as biomarkers in combination with AFP testing to obtain a better sensitivity than AFP alone [93]. Yin et al. found that miR-199a-3p have high specificity and good predictive value in the diagnosis of early-stage alcohol-related HCC cases [100]. Zhan et al. found that patients with high levels of circulating miR-210 are resistant to trans-arterial chemoembolization treatment and have generally poor survival [101]. The levels of circulating miR-106b showed high sensitivity and specificity in differentiating HCC patients from chronic liver diseases or healthy subjects, denoting its clinical relevance [12].

## 6. Long Non-Coding RNA

Long non-coding RNAs (lncRNA) have been defined as transcripts longer than 200 nucleotides that are not translated into proteins and are largely expressed in various tissues [102]. They are also involved in multiple tumour processes including proliferation, apoptosis, invasion and metastasis through chromatin remodelling, epigenetic modifications, and gene regulation. Many previous studies showed that lncRNAs might be used as biomarkers in cancers [11]. Among these, the long intergenic non-protein coding RNA 974 (Linc00974) has been shown to be increased in the serum of HCC patients in comparison to the cytokeratin 19 fragment (CYFRA 21-1) and is useful as a tumour marker to improve the prognosis of HCC patients [103]. In vitro studies showed that Linc00974 causes proliferation and metastasis of HCC cells by interacting with keratin 19 (KRT19) [103]. In addition, the overexpression of lncRNA SPRY4-IT1 has been shown to promote tumour cell proliferation and invasion through the activation of the histone-lysine N-methyltransferase enzyme EZH2 [104]. Accordingly, Jing et al. observed that SPRY4-IT1 levels were significantly upregulated in HCC biopsies compared to the adjacent non-tumour tissues and that the amount of SPRY4-IT1 was significantly higher in the plasma collected in pre-surgery compared to that withdrawn in post-surgery [105]. The lncRNA MALAT1 has been demonstrated to regulate Zinc finger E-box-binding homeobox 1 (ZEB1) expression, promoting HCC development [106]. The evaluation of MALAT1 in peripheral blood and HCC tissues showed that there was a progressive and significant increase of MALAT1 levels in the plasma of patients with increasing severity of disease. On the other hand, plasma MALAT1 levels were significantly low in HCC patients with hepatitis B infection [106]. The circulating lncRNA-CTBP has been shown to have high sensitivity and specificity for discriminating HCC from healthy controls and from cirrhotic patients [107]. Weidong et al. identified three circulating lncRNAs, LINC00152, RP11-160H22.5 and XLOC014172, which, combined with the dosage of AFP, could be potential biomarkers of HCC development both in cirrhotic patients and healthy subjects [108].

## 7. Extracellular Vesicles

Extracellular vesicles are membrane-derived structures, released by cells into their microenvironment, which are classified into exosomes, microvesicles and apoptotic bodies, based on their biogenesis, size, and membrane markers [109]. Exosomes are the smallest subtype, with a diameter of 100–150 nm, and are formed by the fusion of multivesicular bodies and the plasma membrane [14]. Microvesicles have a larger diameter, approximately of 100–1000 nm, and derive from the cell membrane. Apoptotic bodies have the largest diameter, ranging from 1 to 5 μm, and are formed by the aggregation of apoptotic cells [14,110]. Secretion of extracellular vesicles in the body fluids is a common mechanism of cell homeostasis, thus the vesicles content can reflect the disease-associated cellular changes [13]. An enhancement of extracellular vesicle secretion is frequently observed in the serum of patients affected by alcoholic liver disease or by early stage fibrosis associated with chronic HBV or HCV infection. One of the molecules highly enriched within the extracellular vesicles released by HCC cells is the lncRNA TUC339 [111]. Moreover, cirrhotic patients with chronic HBV or HCV infection have an increased amount of Annexin V+, EpCAM+, ASGPR1+ and CD133+ microvesicles [112]. The number CD4+ and CD8+ microvesicles has also been found to increase in patients with liver diseases due to chronic inflammation and elevated number of T-cells in the injured liver [113]. Since multiple diseases are associated with the activation of inflammatory cells, the quantification of inflammatory cell-derived extracellular vesicles is not specific to liver pathology. However, the detection of asialoglycoprotein receptor 1 (ASGPR1), a hepatocyte-specific receptor, of EpCAM/CD133, markers of liver progenitor cells, and of cytokeratin-18 (CK18), a marker of hepatocytes and cholangiocytes, could help to define the hepatic origin of such vesicles [114].

Exosomes contain a wide range of biological molecules, including proteins, lipids and nucleic acids, which are markers of tumour onset and progression [19]. Tumour cells release numerous exosomes that are involved in intercellular communication, angiogenesis, metastasis, drug and radiotherapy resistance [115]. Kogure et al. identified 134 different types of miRNAs in Hep3B cell line‑derived exosomes and found 55 miRNAs were over expressed more than 4-fold in the exosomes compared with the donor cells [116]. The expression levels of 25 of these miRNAs were increased up to 166-fold, 30 miRNAs were decreased up to 113-fold and importantly, 11 miRNAs were only detected in exosomes [116]. Wei et al. identified nine miRNAs differentially expressed in the SMMC-7721 liver cancer cell line expressing VPS4, a protein involved in endosomal transport, and derived exosomes. Particularly, six tumour suppressor miRNAs (miR-122-5p, miR-33a-5p, miR-34a-5p, miR-193a-3p, miR-16-5p, and miR-29b-3p) were significantly up-regulated in exosomes secreted by SMMC-7721 expressing Vps4 versus those produced by SMMC-7721 negative forVps4 [117].

Exosomes have shown to vehicle miRNAs into cells and to alter biological functions by targeting specific genes. Lou et al. found that miR-122 could be transported to HCC cells via exosomes and could regulate the target genes resulting in the improvement of HCC cell sensitivity to chemotherapeutic drugs [118]. Exosomal miR-718 has shown to regulate the homeobox B8 gene expression and to inhibit the differentiation of liver HCC cells [119]. Patients with low numbers of exosomes positive for miR-718 in the serum showed higher probability of tumour recurrence after liver transplantation [119]. Moreover exosomal miRNA content could be useful for the differential diagnosis of liver diseases. In fact, the number of exosomes containing miR-18a, miR-221, miR-222 and miR-224 in the serum of patients with HCC has been found to be significantly higher than that in patients with hepatitis and cirrhosis, whereas the presence of miR-101, miR-106b, miR-122 and miR-195 was found to be significantly reduced in HCC [120]. Shi et al. observed reduced levels of exosomal miR-638 in the serum of HCC patients and a negative association with tumour size, vascular infiltration, TNM stage and poor prognosis [121].

Sohn et al. observed that levels of exosomal miR-18a, miR-221, miR-222 and miR-224 in the serum were significantly higher in patients with HCC than those with chronic hepatitis B or with cirrhosis [120].

Wang et al. [122] analyzed the expression level of exosomal miR-21 in the serum and found significantly higher levels in patients with HCC than in those with chronic hepatitis or healthy volunteers. High levels of miR-21 correlated with cirrhosis and advanced tumour stages. Interestingly, they found high levels of miR-21 both in sera and in exosomes; however, exosomal miR-21 expression showed better sensitivity compared to the circulating free molecules [122]. Exosomal miR-665 levels have been found to be significantly over expressed in tumours with large size (>5 cm), local invasion, advanced clinical stage (stage III/IV) and reduced survival [123]. The expression of miR-939, miR-595 and miR-519d was shown to differentiate cirrhotic patients with and without HCC while miR-939 and miR-595 have been shown to be independent predicting factors for HCC [124].

Exosomal miRNAs are emerging as mediators of the interaction between mast cells and tumour cells. Xiong et al. observed that mast cells are able to block HCC cell metastasis by inhibiting the ERK1/2 pathway through the transfer of the exosomal miRNAs into HCC cells, thus providing new insights for the biological therapy of HCV-related HCC [125].

Exosomes have the potential to be employed in target therapy. In fact, the transfer of miR-142 and miR-223 from human macrophages to liver cancer cells by exosomes has been shown to inhibit the proliferation of tumour cells [126]. Moreover, Ma et al. showed that bone marrow-derived mesenchymal stem cells showed significant anti-tumour activity after their sensitization with HCC cell-derived exosomes and inhibited the proliferation of HCC cells, suggesting that sensitization with cancer cell-derived exosomes may be a novel therapeutic strategy [1].

## 8. Conclusions

Several genetic and epigenetic alterations have shown to contribute to tumour development and progression. During neoplastic process, tumour heterogeneity progressively increases, making it extremely difficult to obtain good response to therapeutic treatments. Early diagnosis and dynamic tumour monitoring represent crucial factors to improve the clinical outcome of malignant tumours including HCC. cfDNA, circulating miRNA and epigenetic alterations are a good source of information for tumour diagnosis. Although HCC driver mutations, such as those in TERT promoter, CTNNB1 and TP53 genes, have been widely observed in tissue biopsies, they have been rarely found in cfDNA probably due to the low fraction of circulating mutated molecules or to the lack in sensitivity of most methodologies. Pre-analytical parameters, such as blood storage and processing, also affect cfDNA integrity and recovery yield [36].

Recently, the development of new technologies such as ddPCR, able to detect one mutant copy in a background of 20,000 wild type molecules, is opening new perspectives in the detection of mutant cfDNA [127,128]. The ddPCR method has wide employment also in miRNA detection since it allows absolute miRNA quantification and is not affected by variations caused by samples and PCR amplification efficiency [127].

Some molecular alterations, such as miR-122 and let-7 expression or RASSF1A hypermethylation, provided good diagnostic results when used alone or in combination with AFP dosage [17,57,89]. Conversely, miR-21 levels [86,93,94,95] and cfDNA integrity [12,42,43,57] show contrasting trends and underlie the need for further investigations.

Although the number of studies evaluating biomarkers in liquid biopsies as diagnostic tools for cancer detection is progressively increasing, very few of them have demonstrated solid diagnostic performance. The Early Detection Research Network, administered by the Cancer Biomarkers Research Group in the Division of Cancer Prevention of the US National Cancer Institute, proposed that the development of biomarkers for cancer diagnosis must undergo five phases, but most of the studies are still in the early phases [129].

Standardization among different laboratories in collecting, storage and analytic methods is a key factor to ensure consistency in clinical application. All the genetic and epigenetic alterations proposed as good tumour markers by the studies described above need further analyses on larger cohorts in order to validate them as HCC diagnostic and prognostic biomarkers. It is likely that in the future some of these biomarkers will be employed, alone or in combination with other already established assays (i.e., AFP), to improve the accuracy in the diagnosis of the medical practice.

## Figures and Tables

**Table 1:** Summary of published articles retrieved from Pubmed on the role of somatic mutations and methylation in non-invasive diagnosis in liver cancer.

DNA Alterations	Gene	Tissue Biopsies N Cases (%)	CfDNA N Cases (%)	Method ^2^	Ref.
Single nucleotide mutations	CTNNB1	0	6/48 (12.5)	ddPCR	[25]
	CTNNB1	11/41 (26.8)	4/41 (9.7)	MiSeq	[26]
	TERT promoter	5/41 (12.2)	11/48 (22.9)	ddPCR	[25]
	TERT promoter	29/41 (70.7)	2/41 (4.9)	MiSeq	[26]
	TP53	1/41 (2.4)	7/48 (14.6)	ddPCR	[25]
	TP53	27/41 (65.8)	2/41 (4.9)	MiSeq	[26]
Hypermethylation	APC	NA ^1^	49/72 (68.1)	MSRE-qPCR	[27]
	APC	NA	36/98 (36.7)	Methylight	[17]
	BVES	NA	29/98 (29.6)	Methylight	[17]
	ELF	22/34 (64.7)	18/31 (58.1)	MSP	[28]
	GSTP1	NA	40/72 (55.6)	MSRE-qPCR	[27]
	GSTP1	23/34 (67.6)	12/31 (38.7)	MSP	[28]
	GSTP1	23/26 (88.5)	16/32 (50.0)	MSP	[29]
	GSTP1	NA	17/98 (17.3)	Methylight	[17]
	HOXA9	NA	20/98 (20.4)	Methylight	[17]
	P16	16/22 (72.7)	13/22 (59.1)	MSP	[30]
	P16	25/34 (73.5)	13/31 (41.9)	MSP	[28]
	RASSF1A	5/5 (100)	59/63 (93.6)	MSRE, RT-PCR	[31]
	RASSF1A	NA	51/98 (52.0)	Methylight	[17]
	RASSF1A	NA	47/72 (65.3)	MSRE-qPCR	[27]
	RASSF1A	32/34 (94.1)	16/31 (51.6)	MSP	[28]
	RASSF1A	NA	77/105 (73.3)	MSP	[32]
	RASSF1A	37/40 (92.5)	17/40 (42.5)	MSP	[33]
	SFRP1	NA	40/72 (55.6)	MSRE-qPCR	[27]
	SOCS3	23/48 (47.9)	34/119 (28.6)	MSP	[34]
	TGR5	NA	77/160 (48.1)	MSP	[35]
	TIMP3	NA	11/98 (11.2)	Methylight	[17]
Hypomethylation	LINE-1	NA	70/105 (66.7)	MSP	[32]

^1^ NA, information not available in the article; ^2^ ddPCR = digital droplet PCR; MiSeq = next generation sequencing method; MSRE = methylation sensitive restriction enzyme digestion; MSP = methylation specific PCR; Methylight = multiplex PCR assay; qPCR = quantitative PCR; RT-PCR = Real Time PCR.
